# Proposed benefits of albumin from the ALBIOS trial: a dose of insane belief

**DOI:** 10.1186/s13054-014-0510-4

**Published:** 2014-09-26

**Authors:** Pietro Caironi, Luciano Gattinoni

**Affiliations:** Dipartimento di Fisiopatologia Medico-Chirurgica e dei Trapianti, Fondazione IRCCS Ca’ Granda – Ospedale Maggiore Policlinico, Università degli Studi di Milano, Via F Sforza 35, 20122 Milan, Italy; Dipartimento di Anestesia, Rianimazione ed Emergenza Urgenza, Fondazione IRCCS Ca’ Granda – Ospedale Maggiore Policlinico, Via F Sforza 35, 20122 Milan, Italy

**Keywords:** ■■■

We read with interest the editorial by Flannery and colleagues [1], who proposed ‘a dose of healthy skepticism’ toward the findings of our Albumin Italian Outcome Sepsis (ALBIOS) trial [2]. Although we feel uncertain about where such ‘health’ may come from, we consider a few extra words necessary. First, the rationale of the subgroup analysis of 90-day mortality on septic shock was based on the Kaplan-Meier estimates for 90-day survivals of the entire study population, which tended to separate between groups from day 30. Although this observation was unexpected, it indicates the potential beneficial effects of ancillary functions of albumin, besides its oncotic property [3–5]. When patients with shock as defined in a broader view (with cardiovascular Sequential Organ Failure Assessment score of 1, 3, or 4; n = 1,303) were considered, the beneficial effect of albumin on 90-day survival persisted (relative risk (RR) 0.88, 95% confidence interval (CI) 0.78 to 0.99, *P* = 0.03) even after adjustment for clinically relevant variables (RR 0.88, 95% CI 0.78 to 1.00, *P* = 0.049). Second, we agree with the authors on the potential biases of an open-label study. Nonetheless, we believe that the shorter time to suspension of vasopressors observed in the albumin as compared with the crystalloid group is a solid finding, as supported by a higher or equal mean arterial pressure over the study observed in the former as compared with the latter group [2]. In conclusion, the available data suggest not only that albumin may be a safe alternative to crystalloids in patients with severe sepsis but that it may be beneficial in those with shock [6]. Further studies are warranted to confirm this hypothesis.

## Abbreviations

Cl: Confidence interval
RR: Relative risk
